# Current practice in ionizing-radiation health examinations at occupational health institutions in Japan: a nationwide questionnaire survey

**DOI:** 10.1093/joccuh/uiag049

**Published:** 2026-09-01

**Authors:** Hiroko Kitamura, Tatsuo Nagata, Takashi Moritake

**Affiliations:** National Institutes for Quantum Science and Technology (QST), 4-9-1 Anagawa, Inage-ku, Chiba-city, Chiba 263-8555, Japan; Occupational Health Training Center, University of Occupational and Environmental Health, 1-1 Iseigaoka, Yahatanishi-ku, Kitakyushu-city, Fukuoka, 807-8555, Japan; Department of Ophthalmology, University of Occupational and Environmental Health, 1-1 Iseigaoka, Yahatanishi-ku, Kitakyushu-city, Fukuoka, 807-8555, Japan; National Institutes for Quantum Science and Technology (QST), 4-9-1 Anagawa, Inage-ku, Chiba-city, Chiba 263-8555, Japan

**Keywords:** occupational health, ionizing-radiation, health surveillance, questionnaire survey, radiation protection

## Abstract

**Objectives:**

To characterize the implementation of ionizing-radiation health examinations at occupational health institutions in Japan and examine how these practices are implemented and interpreted within the current legal framework. We also identified operational factors that may influence their contribution to evaluating radiation-related health effects, fitness-for-work judgments, and occupational radiation protection.

**Methods:**

A cross-sectional online questionnaire survey was conducted from January 28 to February 18, 2025, among member institutions listed in the National Federation of Industrial Health Organization directory. Invitations were mailed to 173 institutions, each designating a respondent knowledgeable about examination implementation. Descriptive results were summarized using item-specific denominators and stratified by annual examination volume.

**Results:**

In total, 90 institutions responded, of which 89 provided ionizing-radiation health examinations. Exposure history was primarily obtained through worker self-report, with limited use of employer documentation or dose records. Lens examinations mainly relied on visual inspection or penlight examination; slit-lamp examination was rare, and retro-illumination imaging was not reported. Skin examinations almost exclusively used unaided visual inspection. Institutions differed in diagnostic classification frameworks and whether personal dose information informed judgment.

**Conclusions:**

Implementation varied across institutions, particularly in exposure assessment and the use of personal dose information in clinical judgment. This heterogeneity may limit not only the interpretability of examination results but also their contribution to evaluating radiation-related health effects, fitness-for-work judgments, and occupational radiation protection. These findings highlight the importance of improving the availability and integration of exposure-related information within existing examination processes, particularly in predominantly low-dose occupational environments.

## Teaser

**Key points**
 

- **What is already known on this topic:** In Japan, ionizing-radiation health examinations are legally required, but operational guidance on exposure history verification, examination techniques, and clinical judgment is limited. National statistics aggregate examinees and individuals with abnormal findings, but interpretation is constrained by nonstandardized thresholds and reporting practices.
- **What this study adds:** A nationwide survey of occupational health institutions demonstrated frequent reliance on worker self-report for exposure history and symptoms, with variable use of personal dose information in diagnosis classification. The findings suggest operational gaps that may influence how ionizing-radiation health examinations contribute to the evaluation of radiation-related health effects, fitness-for-work judgments, and occupational radiation protection.
- **How this study might affect research, practice, or policy:** Interpretation of ionizing-radiation health examinations may benefit from considering their regulatory and institutional context, particularly in predominantly low-dose occupational settings. Improving the integration of exposure information, clinical judgment, and occupational radiation protection activities may strengthen the contribution of health examinations to occupational health surveillance without increasing operational burden.

## 1. Introduction

In Japan, employers are legally required to provide ionizing-radiation health examinations for workers who routinely engage in radiation work and enter controlled areas.^1^ Regulations enumerate examination items but do not specify operational details, such as exposure history verification, techniques for lens and skin examinations, or criteria used by examining physicians to judge findings. Moreover, although national aggregates of examinees and “individuals with abnormal findings” are compiled from statutory reports, nonstandard reporting thresholds and causal attribution limit interpretability.

These institution-level discretionary spaces—how information is collected, which clinical techniques are applied, and how results are judged and reported—may affect the effectiveness (early detection of radiation-related disorders) and efficiency (appropriate use of resources) of the system. Recent policy discussion in Japan^2^ emphasizes strengthening dose management and considering risk-based operation^3^; nonetheless implementation at occupational health institutions has not been systematically described. Evidence is scarce on how exposure history and dose information are obtained and used, which techniques are applied for lens (cataract) and skin assessments, and how physicians judge and report findings to employers.

### 1.1 Objectives

The objectives of this study were to characterize the implementation of ionizing-radiation health examinations at occupational health institutions in Japan and analyze how examination items are selected, conducted, and interpreted in relation to exposure information in routine practice. In addition, this study aimed to identify operational gaps that may limit the extent to which ionizing radiation health examinations contribute to: (1) the evaluation of potential radiation-related health effects, (2) judgments regarding fitness for work, and (3) the advancement of occupational radiation protection, particularly in predominantly low-dose occupational settings.

## 2. Materials and methods

### 2.1 Study design and setting

This cross-sectional study used data from an online questionnaire survey conducted from January 28 to February 18, 2025 among member institutions listed in the National Federation of Industrial Health Organization (Zeneiren) directory^4^ (hereinafter “member institutions”). These institutions primarily provide workplace health services such as health examinations, health guidance, and work environment measurements. In 2023, member institutions conducted 157 659 ionizing-radiation health examinations,^5^ accounting for 41.6% of examinees reported nationally (378 911).^6^

### 2.2 Participants

On January 24, 2025, invitations containing URLs/QR codes to the study information page and online questionnaire were mailed to 173 member institutions. Each institution was asked to designate a respondent familiar with the implementation of ionizing-radiation examinations at the institution. Responses were received from 90 institutions (response rate: 52.0%). The institution was the intended unit of analysis. To ensure anonymity, institution identifiers were not collected. Prior to analysis, all submitted responses were screened for exact duplicates, and none were identified. Because identifiers were not collected, multiple submissions from the same institution could not be ruled out; however, each submission was treated as representing a separate institution.

### 2.3 Pilot testing

The questionnaire was pilot tested among occupational physicians at health examination providers to assess clarity, feasibility, and content coverage. On the basis of feedback, wording was refined, and pilot responses were excluded; only data collected after the revision were analyzed.

### 2.4 Questionnaire and variables

Items covered comprised: (1) institutional characteristics, including annual volumes of general and ionizing-radiation health examinations and respondent role; (2) information regarding whether the institution provides ionizing-radiation health examinations; (3) methods for ascertaining exposure history and, among exposed workers, workplace, task, duration, radiation-related disorders, and symptoms; (4) lens (cataract) and skin examination techniques and interpretation; and (5) diagnostic classification frameworks and use of personal dose information at the clinical judgment stage.

Laboratory tests that are externally quality-assured nationwide, such as white blood cell count, red blood cell count, and hemoglobin or hematocrit, were not examined in detail. In the fiscal year 2024, 161 of 173 member institutions participated in the quality-assurance survey, and all received the highest evaluation category of “Excellent.”^7^ Twelve institutions did not participate due to business circumstances. Figure S1 presents the flow of ionizing-radiation health examinations.

### 2.5 Handling missing data

Analyses were descriptive using item-specific denominators, defined as the number of valid responses per item. “Unknown” responses were analyzed as a separate category, and no imputation was performed. Denominators were reported for each table and figure because item nonresponse varied across questions.

### 2.6 Data analysis

Institutions were categorized by annual ionizing-radiation examination volume as ≤5000 (small), 5001-10 000 (medium), or ≥10 001 (large). Stratified summaries were presented using contingency tables with row percentages. Results were also stratified by annual general health examinations (≤10 000 (small), 10 001-50 000 (medium), or ≥50 001 (large)). Because many tables contained sparse cells, particularly in the largest stratum, analyses were interpreted descriptively without formal hypothesis testing.

### 2.7 Ethics

The survey collected no personal or institutional identifiers. The institutional ethics committee of the University of Occupational and Environmental Health, Japan, determined that formal review was not required.

## 3. Results

### 3.1 Respondents and eligible institutions

Of the 90 responding institutions, 89 (98.9%) provided ionizing-radiation health examinations. Respondents were most commonly administrative staff responsible for health examinations (41/90, 45.6%), radiological technologists (18/90, 20.0%), and administrative managers (14/90, 15.6%) (Table 1). Subsequent analyses were restricted to the 89 institutions providing ionizing-radiation health examinations.

**Table 1:** Respondent roles at participating institutions (*N* = 90).

Category	Role	Institutions, *n* (%)
**Administrative staff**	Administrative manager	14 (15.6)
	Administrative staff responsible for health examinations	41 (45.6)
	Other administrative staff (not responsible for health examinations)	7 (7.8)
	Sales staff for health examinations	1 (1.1)
**Healthcare professionals/technical staff**	Physician	2 (2.2)
	Nurse	3 (3.3)
	Public health nurse	1 (1.1)
	Radiological technologist	18 (20.0)
	Medical laboratory technologist	1 (1.1)
	Technical staff for health examinations (unspecified)	1 (1.1)
**Other (unclassifiable)**	Director of the in-facility examination department	1 (1.1)

### 3.2 Institutional scale

Based on annual ionizing-radiation examination volume, most institutions were small (78, 87.6%), with fewer classified as medium (8, 9.0%) or large (3, 3.4%). By contrast, according to general health examination volume, most institutions were large (49, 55.1%), followed by medium (28, 31.5%) and small (12, 13.5%).

### 3.3 Ascertainment of exposure history and related assessments

Exposure history was most commonly ascertained through worker self-report (47/89, 52.8%), followed by reliance on employer assessment without institutional involvement (22/89, 24.7%). Other approaches included the use of employer-provided dose information since the previous examination (10/89, 11.2%) or employer-provided information on work location or task (9/89, 10.1%); 1 institution reported other methods (1/89, 1.1%).

Among workers with exposure history, workplace, task, and duration were primarily collected through worker self-report (61/89, 68.5%). Smaller proportions relied on employer-compiled information (12/89, 13.5%), whereas 16 institutions (18.0%) reported no involvement. Radiation-related disorders were predominantly assessed by worker self-report (64/89, 71.9%), with limited reliance on employer information (9/89, 10.1%) or clinical judgment at interview (3/89, 3.4%); 13 institutions selected “unknown” (14.6%). Symptoms were also assessed predominantly by worker self-report (75/89, 84.3%), whereas few institutions reported no involvement (7.9%), clinical judgment (2.2%), or “unknown” (5.6%). Scale-stratified distributions by annual ionizing-radiation examination volume are outlined in Table 2.

**Table 2:** Methods used to ascertain radiation exposure history and related details, stratified by annual volume of ionizing-radiation health examinations.

Assessment item/method	Small (*N* = 78)	Medium (*N* = 8)	Large (*N* = 3)	Total (*N* = 89)
	*n* (%)	*n* (%)	*n* (%)	*n* (%)
**Methods used to ascertain whether a worker has an exposure history**				
** Not involved because the employer assesses and evaluates exposure**	19 (24.4)	2 (25.0)	1 (33.3)	22 (24.7)
** Evaluated using dose data since the previous examination obtained from the employer**	9 (11.5)	0 (0)	1 (33.3)	10 (11.2)
** Evaluated using employer-provided information on work location and tasks**	8 (10.3)	1 (12.5)	0 (0)	9 (10.1)
** Evaluated based on worker self-report (eg, questionnaire)**	41 (52.6)	5 (62.5)	1 (33.3)	47 (52.8)
** Other**	1 (1.3)	0 (0)	0 (0)	1 (1.1)
** Unknown**	0 (0)	0 (0)	0 (0)	0 (0)
**Methods used to obtain workplace, task, and duration information among workers with exposure history**				
** Not involved because the employer assesses and evaluates exposure**	14 (17.9)	2 (25.0)	0 (0)	16 (18.0)
** Evaluated using employer-provided information on work location and task location**	10 (12.8)	1 (12.5)	1 (33.3)	12 (13.5)
** Evaluated based on worker self-report (eg, questionnaire)**	54 (69.2)	5 (62.5)	2 (66.7)	61 (68.5)
** Other**	0 (0)	0 (0)	0 (0)	0 (0)
** Unknown**	0 (0)	0 (0)	0 (0)	0 (0)
**Methods used to ascertain history of radiation-related disorder**				
** Not involved because the employer assesses and evaluates exposure**	11 (14.1)	2 (25.0)	0 (0)	13 (14.6)
** Evaluated using employer-provided information on work location and tasks**	7 (9.0)	1 (12.5)	1 (33.3)	9 (10.1)
** Evaluated based on worker self-report (eg, questionnaire)**	57 (73.1)	5 (62.5)	2 (66.7)	64 (71.9)
** Other**	3 (3.8)	0 (0)	0 (0)	3 (3.4)
** Unknown**	0 (0)	0 (0)	0 (0)	0 (0)
**Methods used to ascertain subjective symptoms**				
** Not involved because the employer assesses and evaluates exposure**	5 (6.4)	2 (25.0)	0 (0)	7 (7.9)
** Evaluated using employer-provided information on work location and tasks**	4 (5.1)	0 (0)	0 (0)	4 (4.5)
** Evaluated based on worker self-report (eg, questionnaire)**	66 (84.6)	6 (75.0)	3 (100)	75 (84.3)
** Other**	3 (3.8)	0 (0)	0 (0)	3 (3.4)
** Unknown**	0 (0)	0 (0)	0 (0)	0 (0)

### 3.4 Lens and skin examination methods

For lens (cataract-related) examination, visual inspection by the examining physician (54/89, 60.7%) and penlight examination (26/89, 29.2%) were most commonly reported, whereas fundus (4/89, 4.5%) and slit-lamp examinations by any examiner (2/89, 2.2%) were rare; 3 institutions selected “unknown” (3.4%). No institution reported retro-illumination imaging. Lens findings were usually interpreted by the examining physician (82/89, 92.1%); ophthalmologist interpretation was uncommon (2/89, 2.2%); 5 institutions selected “unknown” (5/89, 5.6%).

For skin examination, almost all institutions reported unaided visual inspection by the examining physician (88/89, 98.9%), with 1 institution selecting “unknown” (1.1%) (Table 3).

**Table 3:** Lens (cataract-related) and skin examination methods by annual volume of ionizing-radiation health examinations.

Examination item/method	Small (*N* = 78)	Medium (*N* = 8)	Large (*N* = 3)	Total (*N* = 89)
	*n* (%)	*n* (%)	*n* (%)	*n* (%)
**Lens (cataract-related) examination techniques**				
** Visual inspection by the examining physician**	46 (59.0)	5 (62.5)	3 (100)	54 (60.7)
** Penlight examination by the examining physician**	23 (29.5)	3 (37.5)	0 (0)	26 (29.2)
** Slit-lamp examination by the examining physician**	1 (1.3)	0 (0)	0 (0)	1 (1.1)
** Retro-illumination imaging by the examining physician**	0 (0)	0 (0)	0 (0)	0 (0)
** Visual inspection by an ophthalmologist for ionizing-radiation or ophthalmic examinations**	0 (0)	0 (0)	0 (0)	0 (0)
** Penlight examination by an ophthalmologist for ionizing-radiation or ophthalmic examinations**	0 (0)	0 (0)	0 (0)	0 (0)
** Slit-lamp examination by an ophthalmologist for ionizing-radiation or ophthalmic examinations**	1 (1.3)	0 (0)	0 (0)	1 (1.1)
** Retro-illumination imaging by an ophthalmologist for ionizing-radiation or ophthalmic examinations**	0 (0)	0 (0)	0 (0)	0 (0)
** Other**	5 (6.4)	0 (0)	0 (0)	5 (5.6)
** Unknown**	2 (2.6)	0 (0)	0 (0)	2 (2.2)
**Interpretation of lens examination findings**				
** Interpreted by the examining physician at each institution**	71 (91.0)	8 (100)	3 (100)	82 (92.1)
** Centrally interpreted by an ophthalmologist using images captured by the examining physician**	0 (0)	0 (0)	0 (0)	0 (0)
** Interpreted by an ophthalmologist for ionizing-radiation or ophthalmic examinations at each institution**	2 (2.6)	0 (0)	0 (0)	2 (2.2)
** Centrally interpreted by an ophthalmologist using images captured by an ophthalmologist for ionizing-radiation or ophthalmic examinations**	0 (0)	0 (0)	0 (0)	0 (0)
** Other**	3 (3.8)	0 (0)	0 (0)	3 (3.4)
** Unknown**	2 (2.6)	0 (0)	0 (0)	2 (2.2)
**Skin examination techniques**				
** Visual inspection by the examining physician**	77 (98.7)	8 (100)	3 (100)	88 (98.9)
** Dermoscopy examination by the examining physician**	0 (0)	0 (0)	0 (0)	0 (0)
** Digital camera for dermatology imaging by an examining physician**	0 (0)	0 (0)	0 (0)	0 (0)
** Visual inspection by a dermatologist for ionizing-radiation or dermatologic examinations**	0 (0)	0 (0)	0 (0)	0 (0)
** Dermoscopy examination by a dermatologist for ionizing-radiation or dermatologic examinations**	0 (0)	0 (0)	0 (0)	0 (0)
** Digital camera for dermatology imaging by a dermatologist for ionizing-radiation or dermatologic examinations**	0 (0)	0 (0)	0 (0)	0 (0)
** Other**	0 (0)	0 (0)	0 (0)	0 (0)
** Unknown**	1 (1.3)	0 (0)	0 (0)	1 (1.1)
**Interpretation of skin examination findings**				
** Interpreted by the examining physician at each institution**	76 (97.4)	8 (100)	3 (100)	87 (97.8)
** Centrally interpreted by a dermatologist using images captured by the examining physician**	0 (0)	0 (0)	0 (0)	0 (0)
** Interpreted by a dermatologist for ionizing-radiation or dermatologic examinations at each institution**	0 (0)	0 (0)	0 (0)	0 (0)
** Centrally interpreted by a dermatologist using images captured by a dermatologist for ionizing-radiation or dermatologic examinations**	0 (0)	0 (0)	0 (0)	0 (0)
** Other**	1 (1.3)	0 (0)	0 (0)	1 (1.1)
** Unknown**	1 (1.3)	0 (0)	0 (0)	1 (1.1)

### 3.5 Diagnosis classification framework and use of personal dose information at the clinical judgment stage

**Figure 1:**
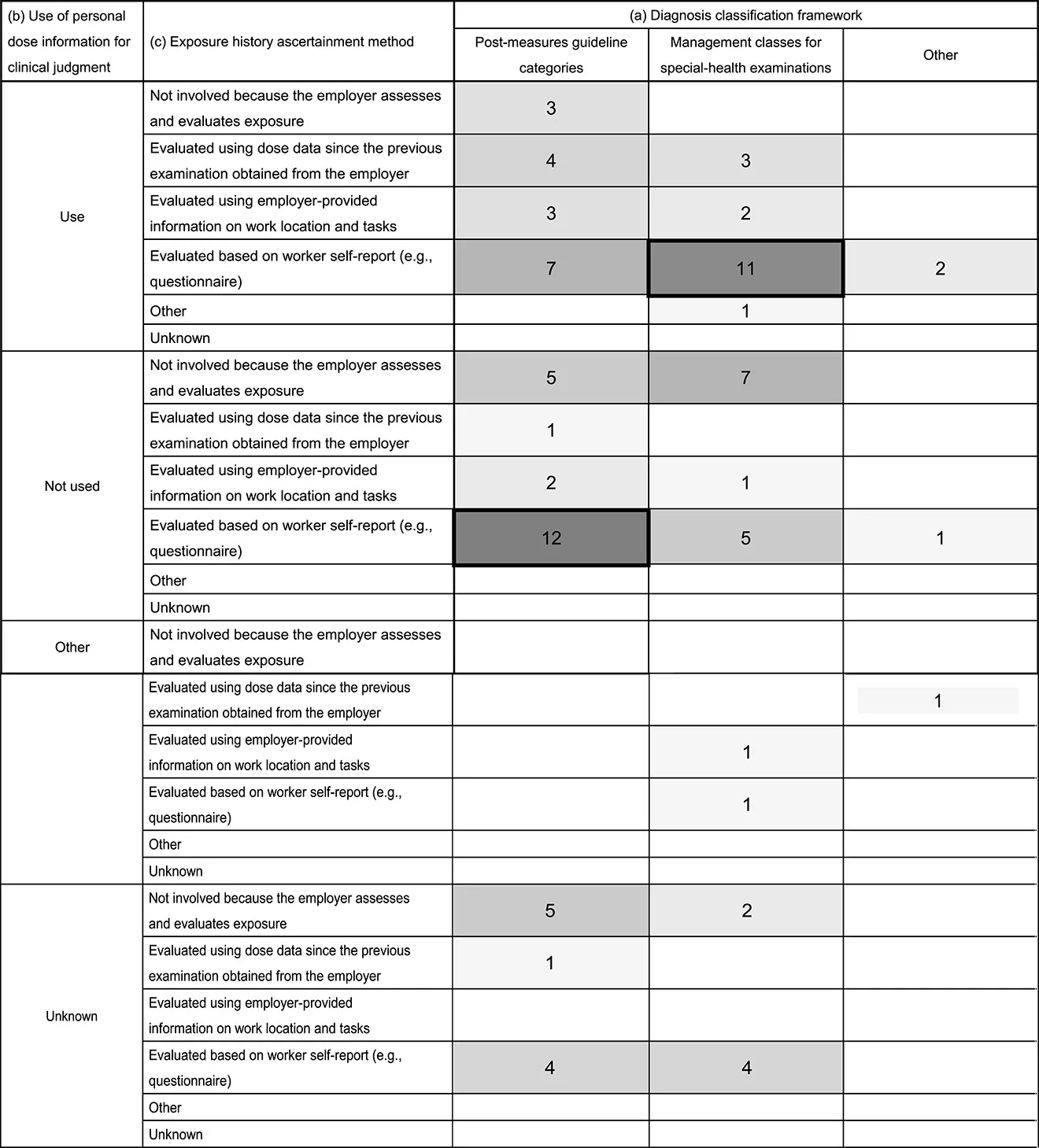
Joint distribution of diagnosis classification framework, use of personal dose information for clinical judgment, and exposure-history ascertainment method. Note: Cells show the number of institutions for each combination of (a) diagnosis classification framework, (b) use of personal dose information for clinical judgment, and (c) exposure-history ascertainment method; darker shading indicates higher counts. Blank cells indicate zero. The response option “no judgment made” was omitted because no institution selected it.

At the institutional level, diagnosis classification most commonly followed the *Guidelines on Measures Employers Should Take Based on Health Checkup Results* (the “post-measures guideline”)^8^ (47/89, 52.8%), followed by *Health Management Based on Health Examination Results* (the “management classes for special-health examinations”)^9^^,^^10^ (38/89, 42.7%); 4 institutions (4.5%) reported other approaches. Personal dose information was used in diagnosis classification by 36 institutions (40.4%), and not used by 34 (38.2%); 3 institutions selected “other” (3.4%) and 16 selected “unknown” (18.0%). Among institutions using management classes for special-health examinations (*n* = 38), 17 (44.7%) used personal dose information, 13 (34.2%) did not, 2 (5.3%) selected “other,” and 6 (15.8%) selected “unknown.” Figure 1 summarizes the joint distribution of the diagnosis classification framework, the use of personal dose information, and the exposure-history ascertainment methods. Overall proportions for the diagnosis classification framework and use of personal dose information, stratified by annual ionizing-radiation examination volume, are delineated in Table 4. Notably, among institutions using management classes for special-health examinations, 21 reported not using personal dose information; within this subgroup, many relied on worker self-report for exposure history or reported no involvement in its ascertainment (Figure 1). Scale-stratified distributions by annual general health examination volume corresponding to Tables 2–4 are shown in Tables S1–S3.

**Table 4:** Method used to judge ionizing-radiation health examinations, by the annual volume of ionizing-radiation health examinations.

Judgment item/category	Small (*N* = 78)	Medium (*N* = 8)	Large (*N* = 3)	Total (*N* = 89)
	*n* (%)	*n* (%)	*n* (%)	*n* (%)
**Diagnosis classification framework for ionizing-radiation health examinations**				
** Post-measures guideline categories**	40 (51.3)	6 (75.0)	1 (33.3)	47 (52.8)
** Management classes for special-health examinations (A/B/C/R/T)^a^**	34 (43.6)	2 (25.0)	2 (66.7)	38 (42.7)
** No judgment made (eg, insufficient dose/work information)**	0 (0)	0 (0)	0 (0)	0 (0)
** Other**	4 (5.1)	0 (0)	0 (0)	4 (4.5)
** Unknown**	0 (0)	0 (0)	0 (0)	0 (0)
**Use of personal dose information for clinical judgment**				
** Used**	32 (41.0)	3 (37.5)	1 (33.3)	36 (40.4)
** Not used**	27 (34.6)	5 (62.5)	2 (66.7)	34 (38.2)
** Other**	3 (3.8)	0 (0)	0 (0)	3 (3.4)
** Unknown**	16 (20.5)	0 (0)	0 (0)	16 (18.0)

^a^A/B/C/R/T indicate Japanese management classes for special-health examinations, which classify findings in relation to the relevant occupational factor: A, no abnormal findings; B, abnormal findings caused or suspected to be caused by the relevant occupational factor, but not classified as C; C, disease caused by the relevant occupational factor; R, disease or abnormal findings not caused by the relevant occupational factor but potentially aggravated by work involving that factor; and T, disease or abnormal findings due to other causes, excluding class R.

## 4. Discussion

Among Zeneiren member institutions in this study, substantial heterogeneity was observed in the implementation of ionizing-radiation health examinations and the interpretation of findings. Although Japanese regulations specify required examination items, they provide limited operational guidance on exposure-history ascertainment, clinical techniques for lens and skin assessment, judgment of findings, and incorporation of personal dose information. Consequently, core elements of examination practice vary across institutions, with possible implications for effectiveness (timely identification of relevant conditions) and efficiency (appropriate allocation of resources and follow-up).

### 4.1 Key gaps highlighted by the survey

First, exposure history and symptom assessment frequently relied on worker self-report rather than employer records or dose information. This reliance may introduce misclassification, such as incomplete or inaccurate exposure histories, and reduce interpretability when institutions judge whether findings plausibly relate to radiation exposure.

Second, lens and skin examinations were dominated by routine practice—feasible approaches with limited sensitivity for consistently documenting early or subtle changes. For lens assessment, most institutions reported visual inspection or penlight examination, whereas slit-lamp examination was rare, and retro-illumination imaging was not reported.^11^ For skin assessment, almost all institutions relied on unaided visual inspection, typically by nondermatologists. These patterns indicate that early lens opacities and subtle skin changes may be difficult to document consistently, particularly in the absence of specialist support and dedicated equipment.

Third, the use of personal dose information for clinical judgment was inconsistent, despite regulations requiring the presentation of dose information since the previous examination. Although withholding dose information during physical examination may reduce observer expectancy bias, dose data remain essential at the judgment stage for assessing temporality and biological plausibility. This tension highlights the need for clearer operational approaches to integrate dose information into clinical judgment, which is further discussed in the following subsection.

### 4.2 Biases that may operate in routine examinations

Several biases may affect how institutional practices translate into reported findings and national aggregates. Observer expectancy (diagnostic suspicion) bias may occur if examining physicians, aware of exposure, apply a low threshold when classifying borderline findings. Detection and documentation differences by institutional scale, such as staffing, equipment availability, and documentation practices, may produce systematic variation in sensitivity and completeness, appearing as between-institution differences even when the underlying burden is similar. Information bias may also arise when exposure history and symptoms are primarily self-reported, including recall error and nondifferential or differential misclassification. These considerations should inform the interpretation of statutory summary statistics and highlight the importance of transparently accounting for variability in examination practices when using aggregated data.

### 4.3 Relationship between examination items and exposure dose

Exposure dose plays a central role^12^ in explaining the biological plausibility of deterministic effects (tissue reactions), including radiation-induced changes in the lens and skin.^13^ From a radiobiological perspective, examination items, methods, and frequency should ideally be selected based not only on exposure dose, but also on dose rate, radiation quality including linear energy transfer, number of exposures (single or repeated), and exposure duration. However, in general occupational settings, collecting detailed radiation-related information for each worker at every health examination is impractical, and therefore, exposure dose is used as a representative indicator.

In Japan, most occupationally exposed workers are managed in very low dose ranges in which deterministic effects are unlikely to occur. According to the 2024 data from the Council on Personal Dosimetry Service,^14^ 94.7% of workers had an annual effective dose of 1 mSv or less. For these individuals, even if exposure conditions remained similar throughout their working lives, the cumulative effective dose would be unlikely to reach levels at which deterministic effects would be of concern (approximately 100 mSv). By contrast, a small proportion of workers may potentially reach a cumulative effective dose around 100 mSv under sustained exposure conditions for decades, including approximately 2700 individuals (0.46%) with annual doses of 5.01-10.00 mSv, and approximately 730 individuals (0.12%) with annual doses of 10.01-20.00 mSv. A very small number (147 individuals, 0.02%) were reported to have annual effective doses exceeding 20 mSv, indicating that occupational exposure risk may not be entirely uniform.

Japanese regulations also allow a lower-intensity approach in low-dose circumstances. In particular, for periodic examinations, when the effective dose in the previous year did not exceed 5 mSv and exceedance is not expected in the current year, a medical interview alone may be considered sufficient at the discretion of the physician overseeing the examination (the regulation does not explicitly designate the physician responsible for this determination). However, implementing this provision in practice requires timely access to individual dose information and clear operational accountability for the omission decision, in addition to the employer’s legal responsibility; these elements may vary across real-world settings.

The results of this study indicate that, in routine practice, legally mandated examination items are often performed using similar examination methods, with limited incorporation of individual exposure dose information or explicit differentiation based on health impact risk. This limited operational linkage between radiation dose and examination practices does not necessarily reflect inadequate practice, and instead highlights structural and practical constraints within the current system. These constraints include limited access to timely dose information at the time of examination, ambiguity in roles and responsibilities between employers and occupational health institutions, and the requirement to conduct health examinations for large numbers of predominantly low-risk workers in order to comply with regulations.

Consequently, the extent to which individual examination items contribute to dose-informed interpretation may be attenuated in routine settings. When the majority of workers are exposed at levels where radiation-related findings are difficult to distinguish from background conditions, and when even relatively higher-exposed workers primarily undergo basic examinations centered on visual inspection, isolating radiation-attributable findings becomes challenging. Balancing the legal and administrative requirements for health examinations with the radiobiological principles underlying dose–effect assessment remains an ongoing challenge for occupational radiation health practice and surveillance.

### 4.4 Functions of ionizing-radiation health examinations

In occupational health practice, ionizing-radiation health examinations can be conceptualized as serving 3 interrelated functions^15^: (1) evaluation of potential health effects related to occupational radiation exposure, (2) support for judgments regarding fitness for work, and (3) strengthening occupational radiation protection.

Interpreting the findings of this survey through these functional perspectives helps clarify what ionizing-radiation health examinations can realistically achieve in a context where low-dose exposure predominates, as well as the operational constraints that may limit the extent to which they can fulfill these roles.

First, with respect to the evaluation of health effects from occupational exposure, a key challenge lies in attributing observed findings to radiation exposure. When most workers are managed at very low dose levels, distinguishing radiation-related findings from changes attributed to background conditions or individual factors becomes inherently difficult. In such contexts, the scope for interpreting examination findings as evidence of radiation-related health effects is limited. As demonstrated in this study, insufficient acquisition and integration of dose information may further narrow the range within which examination results can be meaningfully interpreted in relation to occupational radiation exposure.

Second, from the perspective of fitness for work, health examinations provide information to support judgments about whether workers are able to perform their duties safely and whether continued work may adversely affect their health. However, as shown by the present findings, uniformly applied basic examination methods may fail to capture relevant findings in some cases. In addition, when information on exposure dose or work characteristics is incomplete, fitness-for-work judgments may become largely procedural, potentially limiting their ability to fulfill the intended purposes of safety-related and health-related considerations.

Third, in relation to occupational radiation protection, health examinations can contribute to dose-based management by identifying patterns that may imply potentially concerning exposure situations and supporting workers’ risk awareness that promotes protective behavior. This function presupposes effective linkage between examination processes, clinical judgment, and dose records. When examinations are conducted and reported without clear alignment to exposure information, opportunities for learning and feedback that could strengthen radiation protection practice may be diminished.

Taken together, the central issue is not whether more intensive examinations should be broadly implemented, but rather how the existing system can be aligned with dose-based principles in a manner that remains feasible under real-world operational constraints. This framing also provides a basis for interpreting international approaches that explicitly place worker categorization by dose and structured use of dose records at the core of occupational radiation health management.

### 4.5 International context

Approaches to workers’ health surveillance in occupational radiation protection vary internationally in terms of how health surveillance is structured, including how programs are designed, how medical responsibilities are assigned, and how dose information is integrated into clinical decision-making. These approaches are reflected not only in international guidance but also in regulatory frameworks adopted in Europe^16–20^ and similar regulatory settings. Such differences are not limited to examination items themselves but reflect broader distinctions in regulatory frameworks and professional responsibilities.

In international guidance such as the IAEA Safety Standards on Occupational Radiation Protection (GSG-7),^21^ workers’ health surveillance is framed as a program^21^ based on general principles of occupational health, with the objective of assessing initial and continuing fitness for work. Within this framework, dose records play an important role in informing medical judgment, especially for tasks associated with specific deterministic risks (eg, lens opacities or localized skin disorder). Simultaneously, the guidance explicitly emphasizes that periodic medical examinations should be tailored to the type of work performed and to workers’ age, health status, and relevant habits, and that exposure to radiation should not, in itself, justify more frequent or uniform medical examinations. In this context, radiation exposure and dose information are considered alongside other occupational and individual factors in assessing workers’ health and fitness for work.

In international frameworks, responsibility for workers’ health surveillance is structured across several interconnected levels. At the system level, occupational health services are responsible for ensuring that appropriate health surveillance is provided, including health assessment, record keeping, and counseling. These responsibilities are operationalized through a program for workers’ health surveillance, which defines how health assessments are organized and implemented in practice.

Within this program, occupational physicians are assigned specific medical responsibilities. These include conducting medical examinations of workers, advising on fitness for work, providing clearance for return to work when necessary, and offering medical advice on issues such as contamination and workplace hygiene in collaboration with radiation protection professionals. In practice, this structure often places occupational physicians in a central role in integrating clinical findings with exposure information and task-specific risks.

By contrast, the Japanese system applies legally mandated ionizing-radiation health examinations to a broad population of workers who routinely enter controlled areas, largely independent of individual dose distributions. Therefore, health examinations with broadly similar content are conducted for most workers, reflecting legal requirements rather than graded exposure-based differentiation. Furthermore, these examinations are frequently performed by examining physicians at occupational health institutions, whereas occupational physicians are more commonly involved in interpretation, follow-up, and decisions regarding fitness for work. This represents a distinct division of responsibilities between health examination providers and occupational physicians, which differs from international frameworks, wherein occupational physicians are more directly involved in the conduct of medical surveillance.

From an international perspective, the differences identified in this study—such as the limited use of dose information in clinical judgment and the uniform application of examination items—can be understood as consequences of regulatory and institutional design rather than as deviations from international principles. Thus, the Japanese approach represents a distinct and internally consistent system shaped by its own legal and organizational context. This perspective also helps to contextualize the role and limitations of legally prescribed health examinations in settings where the majority of workers are managed at very low exposure levels.

### 4.6 Implications for occupational health surveillance

The findings of this study have implications for how ionizing-radiation health examinations contribute to occupational health surveillance in Japan.

First, the evaluation of potential radiation-related health effects depends on adequate information regarding exposure history and dose. In this survey, exposure history was frequently obtained through worker self-report, and the integration of dose information into clinical judgment was inconsistent. These practices may limit the ability to assess whether observed findings are plausibly related to occupational radiation exposure. Japanese regulations already permit a lower-intensity examination approach for workers whose previous and anticipated annual effective dose does not exceed 5 mSv. Appropriate implementation of this provision requires access to dose information at the stage of examination planning in order to identify workers for whom examination items may or may not be omitted. Clarifying operational responsibilities for obtaining, providing, and using dose information throughout the examination process may strengthen the contribution of health examinations to the evaluation of radiation-related health effects while supporting risk-based implementation within the existing regulatory framework.

Second, health examinations are expected to support fitness-for-work judgments. In predominantly low-dose occupational settings, this function does not necessarily require increasingly sensitive examinations for all workers. Rather, it depends on the ability to identify situations in which further assessment is warranted. For lens assessment, routine evaluation aimed at identifying visual impairment relevant to work performance may be appropriate for most workers. However, more structured approaches for referral or specialist assessment at defined exposure milestones or other indicators of elevated risk may help balance early identification of potentially relevant findings with the practical constraints of occupational health services. For skin assessment, fitness-for-work judgments require consideration not only of clinical findings alone but also of symptom progression in relation to work activities, work demands, and the extent to which skin conditions may affect a worker’s ability to safely perform assigned tasks. Appropriate interpretation therefore depends on access to relevant exposure information and occupational context, even when causation cannot be attributed to radiation exposure alone.

Third, health examinations may contribute to occupational radiation protection when information obtained through examinations is linked to exposure management and preventive actions. In this survey, however, dose information, examination findings, and reporting systems were not always operationally connected. When these elements function largely independently, opportunities for feedback, learning, and preventive action may be reduced. Strengthening practical linkages between examination processes, exposure information, and occupational radiation protection activities may enhance the contribution of health examinations to dose-based radiation protection without expanding the scope of routine examinations.

Finally, these challenges also affect the interpretability of national surveillance data. The findings of this study suggest that variation in exposure history ascertainment, examination practice, and the use of dose information may influence how examination findings are generated and classified across institutions. Because current statutory reports do not specify which examination items contribute to abnormal findings or stratify results by dose category,^22^ it is difficult to distinguish true health signals from differences in examination practice and reporting. Revising reporting formats to enable item-specific and dose-stratified reporting may improve the interpretability and public health value of surveillance data.

Specific and feasible measures need not involve uniform intensification of examinations. Rather, they may include proportionate procedures that preserve the capacity to escalate assessment when clinically or radiologically warranted. For example, where feasible, institutions could document how exposure history was obtained, whether dose information was available for interpretation, and whether omission of examination items was considered under low-dose conditions. Employers remain legally responsible for implementing ionizing-radiation health examinations, but occupational health institutions may support risk-informed practice by paying attention to whether the information needed for interpretation and follow-up is available. Such measures could help strengthen the practical value of the current system while respecting differences in institutional resources, employer–provider relationships, and worker risk profiles.

Taken together, the findings of this study suggest that improving occupational health surveillance does not necessarily require more intensive examinations. Rather, greater benefit may be achieved by improving the quality, availability, and integration of information used throughout the examination process and by clarifying operational responsibilities within the existing regulatory framework.

### 4.7 Strengths and limitations

This study provides an institution-level description of implementation practices for ionizing-radiation health examinations—an area with limited empirical evidence. By examining how exposure information, examination techniques, and clinical judgment are applied in practice, it identifies operational gaps that can be interpreted within the current legal framework.

This study also has some limitations. First, the sampling frame was restricted to Zeneiren member institutions, limiting generalizability to non–member institutions. Second, nonresponse bias is possible if institutions with differing implementation capacity or interest were more or less likely to respond. Third, institutional identifiers were not collected to ensure anonymity; therefore, multiple submissions from the same institution cannot be excluded, although no exact duplicates were identified. Fourth, responses were self-reported and not verified against documentation or direct observation; thus misclassification cannot be ruled out. Fifth, few medium- and large-scale institutions were included, limiting the precision of scale-stratified descriptions; therefore, patterns were interpreted descriptively without formal hypothesis testing. Finally, this cross-sectional study describes implementation practices but cannot assess clinical effectiveness, costs, or diagnostic performance of examination techniques.

## 5. Conclusions

In a nationwide survey of Zeneiren member institutions, implementation of ionizing-radiation health examinations varied widely in exposure history ascertainment, lens and skin examination techniques, and the use of personal dose information in clinical judgment. This heterogeneity may limit not only the interpretability of examination results but also the extent to which ionizing-radiation health examinations contribute to the evaluation of radiation-related health effects, fitness-for-work judgments, and occupational radiation protection.

These findings indicate that current practices of ionizing-radiation health examinations in Japan are embedded within their regulatory and institutional context. Understanding how examination practices are implemented and interpreted in real-world settings is essential for evaluating their role in occupational health surveillance, particularly in predominantly low-dose occupational environments. Improving the quality, availability, and integration of information throughout the examination process may help strengthen this role while remaining consistent with risk-based principles and operational feasibility.

Further efforts may focus on developing proportionate approaches that maintain the capacity to intensify assessment when clinically or radiologically warranted, while allowing lower-intensity implementation for workers with consistently low exposure. Within the employer-led legal framework, occupational health institutions may also contribute by ensuring, where feasible, that exposure history, dose information, and examination findings are sufficiently connected to support risk-informed interpretation and appropriate follow-up.

## Supplementary Material

Supplementary_material_uiag049

## Data Availability

The data supporting the findings of this study are available from the corresponding author upon reasonable request.
