# Supplementary material for: Current practice in ionizing-radiation health examinations at occupational health institutions in Japan: a nationwide questionnaire survey
Source: J Occup Health. 2026 Sep 1;68(1):uiag049. doi: 10.1093/joccuh/uiag049 (PMC13615524; doi:10.1093/joccuh/uiag049)
Supplement: Supplementary file 1 [file supplementary_material_uiag049.zip › Supplementary_Figure_S1_without_track_uiag049.docx]

Preparation for ionizing-radiation health examinations

[Employer]

Selection of examination workers

Preparation for the dose information since the previous examination

**Conducting Ionizing-radiation health examinations**

**[Occupational Health Institutions]**

**Implementation**

- **Ascertainment of exposure history**
- **Obtaining workplace/task/duration information, radiation-related disorders, and symptoms (among exposed workers)**
- **Hematological examinations**
  - **White blood cell count and white blood cell differential**
  - **Red blood cell count and hemoglobin or hematocrit**
- **Lens (cataract) examination**
- **Skin examination**

**Clinical judgment**

- **Diagnosis classification used by examining physicians**
- **Use of personal dose information**

Submission of statutory reports to the Labour Standards Inspection Office

[Employer]

Follow-up measures based on the results of ionizing-radiation health examination

[Employer]

Consultation for physicians on the results of ionizing-radiation health examination

[Employer]

Fitness for duty assessment

Supplementary Figure S1. Flowchart of ionizing-radiation health examinations
