# Supplementary material for: Current practice in ionizing-radiation health examinations at occupational health institutions in Japan: a nationwide questionnaire survey
Source: J Occup Health. 2026 Sep 1;68(1):uiag049. doi: 10.1093/joccuh/uiag049 (PMC13615524; doi:10.1093/joccuh/uiag049)
Supplement: Supplementary file 1 [file supplementary_material_uiag049.zip › Supplementary_Tables_uiag049.docx]

Supplementary Table S1. Methods used to ascertain radiation exposure history and related details, stratified by annual volume of ionizing-radiation health examinations.

|  | Small (N = 12) | Medium (N = 28) | Large (N = 49) | Total (N = 89) |
| --- | --- | --- | --- | --- |
|  | n (%) | n (%) | n (%) | n (%) |
| Methods used to ascertain whether a worker has an exposure history | | | | |
| Not involved because the employer assesses and evaluates exposure | 5 (41.7) | 3 (10.7) | 14 (28.6) | 22 (24.7) |
| Evaluated using dose data since the previous examination obtained from the employer | 1 (8.3) | 5 (17.9) | 4 (8.2) | 10 (11.2) |
| Evaluated using employer-provided information on work location and tasks | 0 (0) | 5 (17.9) | 4 (8.2) | 9 (10.1) |
| Evaluated based on worker self-report (e.g., questionnaire) | 6 (50.0) | 15 (53.6) | 26 (53.1) | 47 (52.8) |
| Other | 0 (0) | 0 (0) | 1 (2.0) | 1 (1.1) |
| Unknown | 0 (0) | 0 (0) | 0 (0) | 0 (0) |
| Methods used to obtain workplace, task, and duration information among workers with exposure history | | | | |
| Not involved because the employer assesses and evaluates exposure | 1 (8.3) | 3 (10.7) | 12 (24.5) | 16 (18.0) |
| Evaluated using employer-provided information on work location and task location | 1 (8.3) | 5 (17.9) | 6 (12.2) | 12 (13.5) |
| Evaluated based on worker self-report (e.g., questionnaire) | 10 (83.3) | 20 (71.4) | 31 (63.3) | 61 (68.5) |
| Other | 0 (0) | 0 (0) | 0 (0) | 0 (0) |
| Unknown | 0 (0) | 0 (0) | 0 (0) | 0 (0) |
| Methods used to ascertain history of radiation-related disorder | | | | |
| Not involved because the employer assesses and evaluates exposure | 2 (16.7) | 2 (7.1) | 9 (18.4) | 13 (14.6) |
| Evaluated using employer-provided information on work location and tasks | 1 (8.3) | 4 (14.3) | 4 (8.2) | 9 (10.1) |
| Evaluated based on worker self-report (e.g., questionnaire) | 8 (66.7) | 22 (78.6) | 34 (69.4) | 64 (71.9) |
| Other | 1 (8.3) | 0 (0) | 2 (4.1) | 3 (3.4) |
| Unknown | 0 (0) | 0 (0) | 0 (0) | 0 (0) |
| Methods used to ascertain subjective symptoms | | | | |
| Not involved because the employer assesses and evaluates exposure | 1 (8.3) | 2 (7.1) | 4 (8.2) | 7 (7.9) |
| Evaluated using employer-provided information on work location and tasks | 0 (0) | 2 (7.1) | 2 (4.1) | 4 (4.5) |
| Evaluated based on worker self-report (e.g., questionnaire) | 11 (91.7) | 24 (85.7) | 40 (81.6) | 75 (84.3) |
| Other | 0 (0) | 0 (0) | 3 (6.1) | 3 (3.4) |
| Unknown | 0 (0) | 0 (0) | 0 (0) | 0 (0) |

Supplementary Table S2. Lens (cataract-related) and skin examination method, by annual volume of general health examinations.

| Item | Small (N = 12) | Medium (N = 28) | Large (N = 49) | Total (N = 89) |
| --- | --- | --- | --- | --- |
|  | n (%) | n (%) | n (%) | n (%) |
| Lens (cataract-related) examination techniques | | | | |
| Visual inspection by the examining physician | 8 (66.7) | 18 (64.3) | 28 (57.1) | 54 (60.7) |
| Penlight examination by the examining physician | 3 (25.0) | 7 (25.0) | 16 (32.7) | 26 (29.2) |
| Slit-lamp examination by the examining physician | 0 (0) | 1 (3.6) | 0 (0) | 1 (1.1) |
| Retro-illumination imaging by the examining physician | 0 (0) | 0 (0) | 0 (0) | 0 (0) |
| Visual inspection by an ophthalmologist for ionizing-radiation or ophthalmic examinations | 0 (0) | 0 (0) | 0 (0) | 0 (0) |
| Penlight examination by an ophthalmologist for ionizing-radiation or ophthalmic examinations | 0 (0) | 0 (0) | 0 (0) | 0 (0) |
| Slit-lamp examination by an ophthalmologist for ionizing-radiation or ophthalmic examinations | 1 (8.3) | 0 (0) | 0 (0) | 1 (1.1) |
| Retro-illumination imaging by an ophthalmologist for ionizing-radiation or ophthalmic examinations | 0 (0) | 0 (0) | 0 (0) | 0 (0) |
| Other | 0 (0) | 1 (3.6) | 4 (8.2) | 5 (5.6) |
| Unknown | 0 (0) | 1 (3.6) | 1 (2.0) | 2 (2.2) |
| Interpretation of lens examination findings | | | | |
| Interpreted by the examining physician at each institution | 11 (91.7) | 27 (96.4) | 44 (89.8) | 82 (92.1) |
| Centrally interpreted by an ophthalmologist using images captured by the examining physician | 0 (0) | 0 (0) | 0 (0) | 0 (0) |
| Interpreted by an ophthalmologist for ionizing-radiation or ophthalmic examinations at each institution | 1 (8.3) | 0 (0) | 1 (2.0) | 2 (2.2) |
| Centrally interpreted by an ophthalmologist using images captured by an ophthalmologist for ionizing-radiation or ophthalmic examinations | 0 (0) | 0 (0) | 0 (0) | 0 (0) |
| Other | 0 (0) | 0 (0) | 3 (6.1) | 3 (3.4) |
| Unknown | 0 (0) | 1 (3.6) | 1 (2.0) | 2 (2.2) |
| Skin examination techniques |  |  |  |  |
| Visual inspection by the examining physician | 12 (100) | 27 (96.4) | 49 (100) | 88 (98.9) |
| Dermoscopy examination by the examining physician | 0 (0) | 0 (0) | 0 (0) | 0 (0) |
| Digital camera for dermatology imaging by an examining physician | 0 (0) | 0 (0) | 0 (0) | 0 (0) |
| Visual inspection by a dermatologist for ionizing-radiation or dermatologic examinations | 0 (0) | 0 (0) | 0 (0) | 0 (0) |
| Dermoscopy examination by a dermatologist for ionizing-radiation or dermatologic examinations | 0 (0) | 0 (0) | 0 (0) | 0 (0) |
| Digital camera for dermatology imaging by a dermatologist for ionizing-radiation or dermatologic examinations | 0 (0) | 0 (0) | 0 (0) | 0 (0) |
| Other | 0 (0) | 0 (0) | 0 (0) | 0 (0) |
| Unknown | 0 (0) | 1 (3.6) | 0 (0) | 1 (1.1) |
| Interpretation of skin examination findings | | | | |
| Interpreted by the examining physician at each institution | 12 (100) | 28 (100) | 47 (95.9) | 87 (97.8) |
| Centrally interpreted by a dermatologist using images captured by the examining physician | 0 (0) | 0 (0) | 0 (0) | 0 (0) |
| Interpreted by a dermatologist for ionizing-radiation or dermatologic examinations at each institution | 0 (0) | 0 (0) | 0 (0) | 0 (0) |
| Centrally interpreted by a dermatologist using images captured by a dermatologist for ionizing-radiation or dermatologic examinations | 0 (0) | 0 (0) | 0 (0) | 0 (0) |
| Other | 0 (0) | 0 (0) | 1 (2.0) | 1 (1.1) |
| Unknown | 0 (0) | 0 (0) | 1 (2.0) | 1 (1.1) |

Supplementary Table S3. Method used to judge ionizing-radiation health examinations, by annual volume of general health examinations.

| Item | Small (N = 12) | Medium (N = 28) | Large (N = 49) | Total (N = 89) |
| --- | --- | --- | --- | --- |
|  | n (%) | n (%) | n (%) | n (%) |
| Diagnosis classification framework for ionizing-radiation health examinations | | | | |
| Post-measures guideline categories | 10 (83.3) | 19 (67.9) | 18 (36.7) | 47 (52.8) |
| Management classes for special-health examinations (A/B/C/R/T) | 2 (16.7) | 8 (28.6) | 28 (57.1) | 38 (42.7) |
| No judgment made (e.g., insufficient dose/work information) | 0 (0) | 0 (0) | 0 (0) | 0 (0) |
| Other | 0 (0) | 1 (3.6) | 3 (6.1) | 4 (4.5) |
| Unknown | 0 (0) | 0 (0) | 0 (0) | 0 (0) |
| Use of personal dose information for clinical judgment |  |  |  |  |
| Used | 4 (33.3) | 12 (42.9) | 20 (40.8) | 36 (40.4) |
| Not used | 6 (50.0) | 10 (35.7) | 18 (36.7) | 34 (38.2) |
| Other | 0 (0) | 1 (3.6) | 2 (4.1) | 3 (3.4) |
| Unknown | 2 (16.7) | 5 (17.9) | 9 (18.4) | 16 (18.0) |
